# Engaging knowledge users in development of the CONSORT-Equity 2017 reporting guideline: a qualitative study using in-depth interviews

**DOI:** 10.1186/s40900-018-0118-y

**Published:** 2018-10-22

**Authors:** Janet Jull, Mark Petticrew, Elizabeth Kristjansson, Manosila Yoganathan, Jennifer Petkovic, Peter Tugwell, Vivian Welch, the CONSORT-Equity 2017 and Boston Equity Symposium participants, Sarah Baird, Luis Gabriel Cuervo, Sarah Edwards, Regina Greer-Smith, Kjell Arne Johansson, Elizabeth Loder, Anne Lyddiatt, Jessie McGowan, Lawrence Mbuagbaw, Tomas Pantoja, Kevin Pottie, Beverly Shea, Monica Taljaard, Charles Weijer, Howard White, Margaret Whitehead

**Affiliations:** 1 School of Rehabilitation Therapy, Queen’s University, Kingston, Ontario Canada; 2 Ottawa Hospital Research Institute & University of Ottawa, Ottawa, Ontario Canada; 3 Department of Social and Environmental Health Research, Faculty of Public Health and Policy, London School of Hygiene and Tropical Medicine, London, England; 4 Centre for Research on Educational and Community Services, School of Psychology, University of Ottawa, Ottawa, Ontario Canada; 5 Bruyère Research Institute, Bruyère Continuing Care and University of Ottawa, 85 Primrose, Ottawa, Ontario Canada; 6 Department of Medicine, University of Ottawa, Ottawa, Ontario Canada; 7 Methods Centre, Bruyère Research Institute, Bruyère Continuing Care and University of Ottawa, Ottawa, Ontario Canada

**Keywords:** Guidelines, Randomized trials, Health equity, Global health, Interviews, Public-involvement, Patient-involvement, Integrated knowledge translation, CONSORT

## Abstract

**Background:**

Randomized controlled trials (“randomized trials”) can provide evidence to assess the equity impact of an intervention. Decision makers need to know about equity impacts of healthcare interventions so that people get healthcare that is best for them. To better understand the equity impacts of healthcare interventions, a range of people who were potentially the ultimate users of research results were involved in a six-phase project to extend the CONsolidated Standards Of Reporting Trials Statement for health equity (“CONSORT-Equity 2017”). We identified these “knowledge users” as: patients and healthcare researchers, decision makers and providers. This paper reports on one project phase: specifically, a qualitative study designed to integrate the expertise of knowledge users. The experiences and perspectives of knowledge users provided many insights about the reporting of health equity issues in randomized trials. This paper describes key informant interviews with knowledge users that contribute to a better understanding of the effects of an intervention on health equity. Additionally, the paper shows how these insights were used to develop CONSORT-Equity 2017.

**Methods:**

A qualitative study that used the framework analysis method was conducted in collaboration with an international study executive and advisory board team. In-depth semi-structured interviews were conducted with a purposive sample of key informants who: consider the research ethics of, fund, conduct, participate in, publish, or use research evidence generated in randomized trials. Transcripts were coded and analyzed using the seven-stage framework analysis method, and data reported to reflect knowledge user suggestions to develop CONSORT-Equity 2017.

**Results:**

Thirteen key informants, of which three were patients, chose to participate in interviews. Seven themes emerged: “Differentiate the type of trial”, “Prompts for health equity”, “Ethics matter”, “Describe unique research strategies”, “Clarity of reporting”, “Implications of equity for sampling and analysis”, “Think beyond the immediate trial”. The interviews provided direction for the extension of 16 CONSORT-Equity 2017 items.

**Conclusions:**

Key informant interviews were used to identify new concepts that were not generated in our other studies and to develop CONSORT-Equity 2017. We encourage the use of key informant interviews in guideline development to obtain and include the real-life expertise of knowledge users.

## Plain Language Summary

Healthcare decision makers need to know the pros and cons of treatments to address health issues. Health equity is about ways to know about and address unfair differences in health for groups of people. To promote health equity, decision makers need to know if the pros and cons of treatments might differ across groups of people, so that more people can get the healthcare best for them. For this reason a guideline to report research details about health equity was made.

In a study to make the guideline to report research details about health equity, we talked with a range of people who join in or use healthcare research and that include patients, healthcare researchers, healthcare decision makers and healthcare providers. Our research shows that all of these “knowledge users” have expertise to share and that their ideas should be valued. We also wanted to know what knowledge users think are key research details about health equity. We describe talking with a group of 13 knowledge users of which 3 were patients, about what they view as key ways to report research details about health equity. Then, we describe how what was learned in talking with knowledge users was used to make the guideline. We conclude that talking with knowledge users is a way to improve guidelines that report on key research details about health equity so that more people can get the healthcare that is the best for them.

## Background

Evidence-based decision making aims to integrate research evidence with practical knowledge for use by health decision makers, and this can have important implications for healthcare systems and consumers [1]. The promotion and utilization of research findings, in both healthcare and health policy, is a tenet of international policies on research for health [2–4]. Considerable efforts have been made to understand and reduce the key sources of bias in research in order to contribute the best evidence for use in health systems. Randomized controlled trials (“randomized trials”), for example, minimize the risk of bias and are more likely to result in moderate or high certainty evidence than non-randomized studies [5]. Randomized trials can provide evidence on both the desirable and undesirable effects of interventions, as well as the differential effects across subpopulations. Differences in health within or between population groups within society, or health inequalities, are labeled “inequities” when they are considered unfair [6]; that is, when they are potentially avoidable [7]. There is a need to study and report on differences in the effects of interventions for population groups to assist those who make decisions in health systems to improve health equity and to ensure that healthcare consumers receive the best care.

Randomised trials can provide evidence relevant to assessing the equity impact of an intervention. A randomized trial can be classified as “health equity relevant” if it assesses the effects of an intervention on health or on the determinants of health for a population who experience ill-health due to disadvantage defined across one or more factors (“social determinants of health”) [8]. A randomized trial is also health equity relevant if it compares the effectiveness of the intervention across levels of advantage/disadvantage. We defined two types of randomized trials relevant to health equity: 1) randomized trials focused on a population experiencing a disadvantage (e.g. low-income parents), and 2) universal randomized trials where differential effects are assessed (e.g. analysis of effects of programs for low-income vs higher income parents) [8]. Reported evidence from randomized trials on health equity can be incorporated into systematic reviews and review-derived products (policy briefs, overviews, guidance documents) to contribute to a robust evidence base for decision making that considers impacts on health equity across populations.

**Fig. 1:**
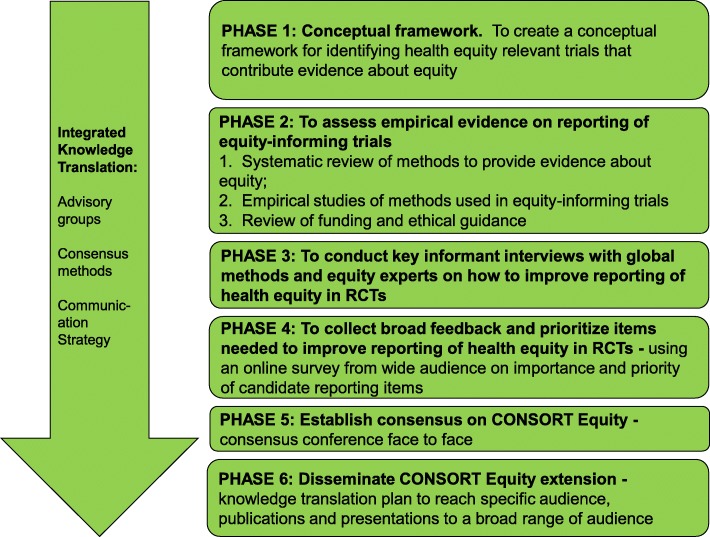
Research Program for CONSORT Equity

The CONsolidated Standards Of Reporting Trials Statement (“CONSORT Statement”) comprises an evidence-based set of recommendations for reporting randomized trials. The CONSORT Statement consists of a 25-item checklist and flow diagram. The checklist focuses on reporting how the trial was designed, analyzed and interpreted, whereas the flow diagram depicts participant progress through the trial processes. Extensions to the CONSORT Statement have been developed for specific issues, such as reporting of cluster randomized trials, harms, pragmatic trials, non-pharmacologic therapies, and social and psychological interventions [9]. As none of the existing extensions of the CONSORT Statement cover the items for reporting needed to assess the effects of an intervention on health equity [10], a program of research was undertaken to develop an extension of the CONSORT Statement to improve reporting of health equity relevant randomized trials [11]. In the development of the program of research, it was identified that there is a need for more active roles of patients and public as part of a group of potential “knowledge users”: individuals directly affected by research and inclusive of those who occupy a range of positions in health systems [12]. The participation of knowledge users in the conceptual development of reporting guidelines, their contributions to an evidence base for patient involvement and their confirmation that what is being reported is of relevance to patients and the public were all identified as important in the work to extend the CONSORT Statement for equity. For this reason, it was defined as critical that the work be done in a way that is inclusive of people for whom the research is meant to ultimately be of use. In other words, to also include those who are, have been or who may become research participants/community collaborators and/or who are knowledge users of the evidence from randomized trials. This paper reports on one study conducted as part of a program of research that aims to integrate knowledge user involvement in the conceptual development of a reporting guideline (Fig. 1).

The extension of the CONSORT Statement for equity (“CONSORT-Equity 2017”) [11] followed the proposed steps for developing consensus-based reporting guidelines described by Moher et al. [13]. Because ensuring the uptake of CONSORT-Equity 2017 is critical to influence reporting of future randomized trials, the decision was made to use an integrated knowledge translation approach which engages knowledge users as partners throughout the process. The aim was to foster thinking about, and inclusion of, a multiplicity of perspectives [11, 14]. A key feature in the development of CONSORT-Equity 2017 has been the involvement of an international and inter-disciplinary study executive (a small group of people who take responsibility for developing and executing the project) and an advisory board [10].

The study executive members represent a range of disciplines including clinical epidemiology, social science, public health, and international development. Study executive members were consulted regularly through planned meetings. Members of the study executive identified the importance of collaboration with knowledge user groups during the development of CONSORT-Equity 2017. We also recruited an international advisory board, being careful to include a broad range of member perspectives, including: patients, journal editors, trialists, bioethicists, clinicians, systematic review authors, policy makers, and funders. These knowledge users are described in greater detail elsewhere [10]. The role of the advisory board membership was agreed upon by study executive and advisory board members: specifically, the advisory board was to participate in a collaborative process, provide content-related support, and bring knowledge, skills and experience to the working group throughout the multiple stages of the research process. The advisory board was facilitated by one researcher (JJ) to ensure communication within and between the advisory board and study executive groups. As well, the inclusion of the advisory board created an opportunity for debate and discussion among the study members, such as the exploration and expansion of concepts related to health equity [8, 10]. Their contributions throughout the steps of the program of research guided the development of CONSORT-Equity 2017.

To ensure knowledge user participation, the study executive and advisory board members decided to adapt the guidelines described by Moher et al, to include interviews with knowledge users as “key informants”; that is, individuals who are potentially the ultimate users of the results of the research. It was important to engage with those who consider the ethics of, fund, conduct, participate in, publish or use the research evidence generated in randomized trials who have a range of perspectives and expertise that would be difficult to capture through an online survey alone. As well, it was important to promote an approach to guideline development that creates opportunities for the inclusion of knowledge users, including patients and the expertise that these groups offer. Differences in culture, training, priorities et cetera create challenges to practicing partnered research that leads to co-creation of knowledge, and we describe our approach to engagement with knowledge users in guideline development. The purpose of interviews with key informants was to elicit detailed knowledge user views on the ways to improve reporting of intervention effects related to health equity in randomized trials. The aim of this manuscript is twofold: (i) to describe key informant views about items for reporting to assess effects of an intervention on health equity, and (ii) to describe how the key informant interviews were used to develop CONSORT-Equity 2017.

## Methods

The details of this study and the development of CONSORT-Equity 2017 are described elsewhere [10, 11]. Key informant interviews are an efficient way to engage with, collect and synthesize an array of views. The Bruyère Research Institute’s Research Ethics Board approved this study in February 2016 (Protocol # M16-15-042). Funded by Canadian Institutes of Health Research (MOP_133556).

### Design

This was a qualitative study using in-depth, semi-structured interviews. Health equity issues span a broad range of disciplinary fields that may have different approaches to reporting randomized trials. We aimed to achieve inter-disciplinary relevance of CONSORT-Equity 2017 as a reporting guideline. Therefore, we sought knowledge user views about how to improve reporting of equity-relevant randomized trials in a way that aligned with the multi-disciplinary, theoretical and ethical approach used in the CONSORT-Equity 2017 study and reported in Table 1 [15]. Key informant interviews were identified as a method acceptable to potential participants and to CONSORT-Equity 2017 team members. The use of key informant interviews created opportunities to engage with participants and obtain potentially new knowledge in the guideline development process. The participant sample size was determined by theoretical saturation, defined as when subsequent interviews contribute no new concepts. We estimated that this would occur at 10–13 interviews [16]. Qualitative key informant interviews were conducted from March 2016 to July 2016 so that results could feed into the later phases of the guideline development process. We then analysed the qualitative data with the framework method of thematic analysis [17]. The framework method involves development of an analytical framework that is then used to organize and index data, and, if needed, to make adjustments to the framework for any additional themes [17]. The study was conducted with iterative feedback from the study executive and advisory board members.

**Table 1:** GRIPP (Guidance for Reporting Involvement of Patients and the Public) 2 Short Form Reporting checklist for study conduct

Section and Topic	Item	Reported on page No.
1. Aim	Report the aim of PPI^a^ in the study	Aim described in Abstracts (see lay and technical), and Background section (para. 4 and 5)
2. Methods	Provide a clear description of the methods used for PPI in the study	Methods section see “Design” and “Setting and participants” section (para. 2,3 in methods)
3. Study results	Outcomes – report the results of PPI in the study, including both positive and negative outcomes	See “Results” section, “Limitations and Strengths” paragraphs that discuss PPI
4. Discussion and conclusions	Outcomes – Comment on the extent to which PPI influenced the study overall. Describe positive and negative effects	See “Discussion” (para. 2,4) and “Limitations and Strengths” section
5. Reflections/critical perspective	Comment critically on the study, reflecting on the things that went well, and those that did not so others can learn from this experience	See “Limitations and Strengths” section

^a^Patient and public involvement

### Setting and participants

We used a purposive sampling approach [18] and identified potential participants for key informant interviews through contacts suggested by the study executive and advisory board members. In order to ensure that participants were from knowledge user groups, we included people who are involved in the ethical approval, funding, conduct, publication, or use of research evidence generated in randomized trials, or who participate in randomized trials. Participants were selected to maximize variation of disciplines and experience (for example, their role in relation to health research, years of experience in the role, familiarity with CONSORT Statement, general region of current work) and with a focus on those who are known to be direct users of CONSORT Statement as a reporting guideline. We faced challenges in identifying knowledge users who are patients and had experience with participation in randomized trials and who might be interested in our study, which was in itself an important observation of the study. Interviews were conducted either in person or with the use of video conference software and were digitally audio-recorded and with notes taken by the researcher during the interviews. The names and identifying characteristics of the study participants were not reported to preserve anonymity.

### Procedure

All interviews were conducted by the first author (JJ) who is experienced in qualitative research and who has played a leadership role in other qualitative studies that involve engagement with a range of knowledge user participants [19, 20]. The lead researcher (JJ) has been involved with the development of CONSORT-Equity 2017: this involvement has been focused on the engagement of knowledge users in the study as the lead of the advisory board and integration of knowledge user views in the CONSORT-Equity 2017 [11]. A semi-structured interview guide based on a previous study conducted with patient participants [19] was developed with feedback from the study executive and advisory board members and pilot-tested. The interview guide was designed to invite feedback on the original CONSORT Statement items as well as to seek new candidate items for inclusion in the proposed version of CONSORT-Equity 2017 (Table 2). Information that introduced study concepts and the aims of the CONSORT-Equity 2017 study was developed and pilot-tested with a range of knowledge user groups and that included patients and members of the public. Written informed consent was sought and obtained from all participants. First, participants were oriented to the study concepts, that is, there was an explanation for the rationale and processes used to develop CONSORT-Equity 2017 as well as how the key informant interviews were anticipated to contribute to the overall study process. Then, participants were provided with a working definition of “health equity” and with examples. Other concepts related to the CONSORT Statement and our work to define health equity relevant randomized trials were clarified [5, 7]. Next, participants were led through a 30 to 60 minute interview with the 25-item CONSORT Statement and asked about the CONSORT Statement in relation to reporting health equity considerations. They were also asked to explain their views. Interviews were conducted with participants until we reached saturation of key concepts [16]. We also conducted three additional interviews to confirm that new concepts did not emerge. The digital audio-recorded interviews were transcribed verbatim, anonymized, and then reviewed by the researcher (JJ) and returned to each key informant participant for member checking so that participants could confirm their transcript for accuracy.

**Table 2:** Questions asked during key informant interviews

Topic	Question	Probes
Experience	Q1. Do you have any experience with reading/using/doing/participating in trials you might consider equity-relevant?	Prompt: Some experience? None? Interest in? Value of? Can you explain your role? When was this?]
Familiarity with checklist use	Q2. Would you explain your experience with CONSORT?	Prompt: Some experience? None? Interest in? Value of? Can you explain your role? When was this?
Feedback on the checklist. [Make sure that the participant has a copy of the checklist in front of them]	Q3. a. Now I will review each section of the CONSORT checklist with you and will ask you about it, okay? For the section titled [title/abstract et cetera – each section of the checklist], is it important that the information on how equity is relevant for the trial is reported for the [title/abstract et cetera]? [This process will be repeated with each section of CONSORT checklist – there are six sections: title/abstract, introduction, methods, results, discussion, other information. There are subsections for each section and it is expected that participants will refer to subsections and issues of particular importance, if needed].	Why? Are there any reasons that it might be challenging to include information on how equity is relevant for a trial in this section? [For each section of the CONSORT checklist]
Perceived use of checklist	Q4. Would you use the CONSORT checklist if it was modified to include equity-relevant considerations?	Why or why not?
Perceived relevance of checklist	Q5. Would you encourage others to use the CONSORT checklist if it was modified to include equity-relevant considerations?	Why or why not?
Additional comments	Q6. Any other comments or suggestions to make the checklist better for reporting equity-relevant trials?	-

### Data analysis

The framework method is a systematic approach to qualitative content analysis of data that identifies similarities and differences, defines relationships, and builds conclusions. In our study, the framework method was selected for analysis of the qualitative interviews as a useful way to explore and link information gathered from an array of interview participants. The framework method was also selected because it is a pragmatic approach: it is useful when multiple researchers in multi-disciplinary teams are working together [17]. The pragmatic feature of the framework method was viewed as an important factor in our research, which included study executive and advisory board members who might not have been familiar with qualitative methods. The framework approach requires leadership from an experienced qualitative researcher, and allows involvement of non-specialists in the analysis process [17]. The transcripts of interviews underwent analysis by two independent reviewers (JJ, MY). JJ led the analysis with the second reviewer (MY) independently confirming the analysis process: all disagreements were reviewed by a third investigator (VW). The seven-stage process of the framework method was used for analysis [17]. These stages were: 1) transcription; 2) familiarization with the interview by reviewers; 3) the generation of initial codes within each transcript interpreted by reviewers as important (e.g. “different types of equity studies”; “don’t make assumptions”; 4) development of a working analytical framework after coding the first few transcripts, and comparison of the labels among the reviewers (JJ, MY) to agree on the set of codes to use in the subsequent transcripts (e.g. “differentiate the type of trial”; “prompts for equity”); 5) application of the analytical framework; 6) inserting data from the remaining transcripts into the framework (further confirmed or adjusted by the second reviewer (MY) to ensure consistency); and 7) interpretation of data so that the characteristics of and differences between the data were identified. The data were reported so that the participant rationales for the comments and/or suggestions for change extend the CONSORT Statement for equity. Two people (JJ, MY) were central to the process and others, directly involved study executive members (EK, JP, MP, PT, VW), had the process described to them, with opportunities for feedback from members of the study executive and advisory board. At the completion of the analysis, findings were reviewed and confirmed with study executive members.

Following analysis, the themes generated during the key informant interviews were used in the development of CONSORT-Equity 2017, and the information used in the subsequent phases (to collect broad feedback and prioritize items needed to improve reporting of health equity in randomized trials, establish consensus on CONSORT-Equity 2017) to develop CONSORT-Equity 2017 [11].

## Results

### Participant characteristics

We interviewed 13 people of which three were patients active in research and so identified as “participant/research collaborator” (Table 3). During the initial recruitment, three potential interviewees declined and all three indicated this was due to lack of time to participate in the interview process. Participants self-identified as having one or more roles in research, with many identifying as having more than one role (for example, “participant/research collaborator” and “researcher” , “researcher” and “journal editor”). Participants reported a mix of research involvement (meaning in the funding, development of, conduct, participation in, dissemination of, or use of outcomes from randomized trials). They also reported a range of experiences with the use of different types of study designs (quantitative, including randomized trials; qualitative; mixed methods; and, systematic reviews). Most participants reported having used or referred to the CONSORT Statement. Participants identified their primary regions of work related to health equity as based in their country of residence and/or internationally.

**Table 3:** Background characteristics of participants (*n*=13)^a^

Characteristics	Frequency
Role in health research	
Funder	1
Ethicist	1
Researcher (includes clinician-researchers)	8
Participant/research collaborator	3
Publisher/journal editor	2
Policy/decision maker	2
Years of experience in role	
1-5 years	2
5-10 years	4
More than 10 years	7
Type of research design	
Quantitative	2
Qualitative	3
Mixed Methods	2
Systematic Reviews	3
All	4
Have used (reported or refer to) CONSORT reporting guidelines	
Yes	9
No	4
Region of current work	
Country of residence	6
International	7

^a^Participants could identify as belonging to more than one category of knowledge user

### Participant reflections on a need for the equity extension for CONSORT

The participants were interested in exploring the potential for the extension of the CONSORT Statement to accommodate consideration of health equity in the reporting of randomized trials. Participant responses to questions were classified into seven themes: “Differentiate the type of trial”, “Prompts for health equity”, “Ethics matter”, “Describe unique research strategies”, “Clarity of reporting”, “Implications of equity for sampling and analysis”, “Think beyond the immediate trial”. The value added by key informant interviews to the extension of the CONSORT Statement for equity is reported in Table 4.

**Table 4:** Value added to the overall development of CONSORT Equity

Category 1: “Differentiate the type of trial” This category contributed to thinking and discussion during the Delphi and consensus meeting about the extension of CONSORT Statement items to report if equity is a major focus in the title (Item 1a) and in the abstract section to consider reporting any research question(s) related to health equity, as well as the results of all planned health equity analyses and the extent and limits of applicability across population characteristics (Items 1b).	
Category 2: “Prompts for health equity” This category contributed to thinking and discussion about how to extend the CONSORT Statement background section so that reporting on the rationale to focus on health equity could be explicit, and also provided the opportunity to state whether or how the objectives of the trial relate to health equity (Items 2a, 2b). For this reason, in the survey and discussions that followed the key informant interviews, concerns about whether and how to extend the CONSORT Statement for equity were considered so that they would be most likely “usable”. Findings helped to guide the broad inclusion of membership in the Delphi survey and invitations to attend the face-to-face consensus meeting. Care was taken to define health equity in trials and examples of “health-equity relevant trials” sought and tested and agreed upon by the CONSORT Equity core team and advisory board.	
Category 3: “Ethics matter” As there are no items related to ethics in the original CONSORT Statement, participant suggestions led to the proposal in the Delphi survey for the addition of a new item (to report details of ethical concern and patient/community engagement) and consideration for extensions of existing items to bring equity considerations forward within the randomized trials (for example, item 6a to report whether items were identified as relevant and important to populations and how it was done). These extension items were affirmed during the Delphi study and then further discussed (and approved) during the consensus meeting.	
Category 4: “Opportunity to describe unique research strategies” During the interviews, most key informants raised concerns about innovative approaches to research that facilitate inclusive randomized trials. For this reason, the extension of the item about randomized trial design to describe aspects of the study design that were chosen to answer equity questions was specifically proposed and then voted upon in the Delphi study. The inclusion of an item to describe unique research strategies was then discussed during the consensus meeting and agreed upon with changes made to ensure there were opportunities to describe/explain unique strategies with description of trial design (item #3a).	
Category 5: “Details are important” This category contributed to thinking and discussion during the Delphi and consensus meeting about the extension of CONSORT Statement items to consider reporting on research methods and the reporting of details on participants and the settings and locations where the data were collected (items 4a, 4b). As well, this contributed to the background information for CONSORT Equity and the explicit use of the PROGRESS Plus framework to guide the thinking about how to describe details on the study as they relate to health equity (item 15).	
Category 6: “Guidance for statistics” Many participants emphasized the benefits of consultation with a biostatistician to help with general barriers to sample size calculations and the lack of evidence about equity-relevant trials and statistical analysis. For this reason, care was taken to include biostatisticians in the Delphi survey and as part of the group membership at the consensus meeting. During the Delphi survey and the consensus meeting there was broad discussion about sample size and the importance of reporting and that focused to be about whether analyses are powered to detect differences (item 7a) as well as about any additional analyses focused on health equity (item 12b) and ancillary analysis (item 18).	
Category 7: “Think beyond the immediate trial” During the Delphi study additional detail for items about harms and the study discussion section were proposed. These were carried forward to the consensus meeting and discussion focused on the need for reporting about whether intervention generated inequities (that is, unintended effects) were assessed, any limitations related to assessing effects on health equity, and reporting of applicability related to the population of interest (Items 19, 20, 21). For, as one participant stated “identifying inequity is the first step but it does not get us further in promoting equitable health if we don’t move past and say “now what?””.	

#### “Differentiate the type of trial”

Most participants emphasized the need to be able to report differently about a focused randomized trial with an explicit and sole focus on assessing intervention effectiveness in a population that experiences a disadvantage (e.g. low income parents) that is linked to poor health in relation to the general population (i.e. leads to health inequity). This is compared to a universal randomized trial in which a sub-analysis has been conducted to assess differential effects between populations (e.g. between low-income children and children from families of higher socioeconomic status). Participants felt that the CONSORT Statement would need to be adjusted to account for these different types of randomized trials to report on equity-related information:

“*[how equity is reported depends upon] if equity is at the heart of what you’re doing either because you’re focusing on a specific subgroup or you’re working in a low and middle income country then….then I don’t know exactly how to say this - but I think the point is that it just depends a bit on how equity fits within your overall narrative”* (Interview #5: researcher, publisher/editor).

*“I have heard of trials…that have conceptualized equity as a sort of first - like a primary outcome – that has been made explicit and in that case you’d definitely want that [focus on reporting health equity with the CONSORT Statement checklist]. But I think that if you are talking about some sort of secondary analysis maybe less so”* (Interview #7: researcher).

#### “Prompts for health equity”

Participants indicated the need to report the features of randomized trials that relate to health equity, and to enhance the CONSORT Statement users’ understandings of equity. All participants discussed the importance of description about how the randomized trial relates to health equity. The re-structuring of the CONSORT Statement so that it is possible to reflect the type of contributions a randomized trial has made to evidence about health equity was identified as important:

*“So they [researchers using the reporting guideline] would really have to define what they [researchers] mean by inequities, how they’re measuring it, and what threshold they’re going to use to say equity has been improved or inequity reduced. Yeah – so that might be challenging because this is a really new, a new field”* (Interview #9: researcher).

Most participants emphasized the need for knowledge user education to prompt critical thinking about health equity (for instance about what is meant by “health equity relevant”, how to identify information related to health equity):

*“People need to be educated on where equity can be found so if they are doing systematic reviews, they can cast a wider net. And then you can re-encourage people – researchers – to actually see that their results are speaking to equity or inequity and that they publish those results separate from other parts of the effectiveness results”* (Interview # 13: policy/decision maker).

#### “Ethics matter”

The majority of the participants emphasized the need for the explicit consideration of ethics within health equity and randomized trials in the extension of the CONSORT Statement. For example, participants expressed concerns about inadvertently perpetuating inequity through the conduct and reporting of randomized trials:

*“This is probably a bit of my ethics background coming in but you want to make sure that when you’re looking at different things like race or gender – that you know there’s a real purpose for it...that there’s a good fit with your question and then I think that needs to be presented in the background, but in a way that you know, people reading it…would not view a certain group more negatively because of that. You don’t want to contribute to ongoing inequity”* (Interview #6: researcher, ethicist).

Participants also described the need to consider contributions to a broader social agenda about health equity:

*“They’re not thinking about what they can do with it so they end up producing absolutely excellent and fantastic RCTs [randomized controlled trials] over a large number of years, it’s almost a careers work for some of them, and they may not actually have even thought what, even in the broadest terms, even when you say well what’s the benefit to society? How is the human race going to benefit from this?”* (Interview #1: policy/decision maker, funder).

In addition, a few participants expressed concern about standard ethical procedures as a barrier to inclusive research conduct:

*“…there’s an assumption that everybody is the same and that things are appropriate for… the white middle class population are going to be appropriate for other communities without any idea that [factors such as] culture, language need to be reflected in research if they’re going to be relevant to the population. Consents drive me crazy. You have a population where most people can’t read or write and…give them a consent form that is so word dense they look at it and assume that they can’t understand it.”*

*“We’re all expected to use the evidence based methods of treatment which is great except that the people we work with would never in a million years have participated in any of the research”* (Interview #4: policy/decision maker, participant/research collaborator)

#### “Describe unique research strategies”

All participants described how research methods could themselves be used to promote equity – for example, through new approaches to research and/or through better reporting of the design and conduct of their research that considers health equity:

*“I think that even to remind people to think about…in terms of developing methodologies or how things are going to be done…to think about it in terms of being strengths based. Excluding people because they cannot read or write or because they don’t have good memory or because they’re not able to generate their own concepts. It does not mean that they cannot participate and it doesn’t mean that they don’t have important things to offer. It just means that we have to get at that information a little bit differently”* (Interview # 4: policy/decision maker, participant/research collaborator)

*“Sometimes people do [consider equity issues in their trial] but they don’t report it, they don’t even realize its important”* (Interview #12: editor)

Many participants acknowledged that unique strategies to consider health equity might be required, using transparent and evidence-based approaches to science and in a way that contributes to build knowledge about health equity in research:

*“So again, speaking largely from sex and gender, equity overall has been, you know, not addressed or not addressed adequately. And...the other overall point and maybe it applies to most is that randomized controlled trials aren’t really designed to talk about context. It’s like trials aren’t really designed to explicate enough about equity, I think”* (Interview # 10: researcher).

*“[Researchers need to know] how to present short simple papers, and include equity focused results without getting carried away, making it lengthy and dragging it on”*

(Interview #5: researcher, editor).

#### “Clarity of reporting”

All participants emphasized the importance of knowing the details on who is – and who is not – included in the study; that is, the participant and study context. They also emphasized that it is important to include and report clearly on populations that are not often included in randomized trial designs (for example, Indigenous people). Participants discussed the need for the CONSORT Statement to be extended to provide clarity of reporting about study populations:

*“Sometimes with the eligibility criteria I find that it isn’t always reported clearly enough. When review authors go to create a systematic review that’s one of the main issues - that authors of trials haven’t reported fully enough. They [authors of trials] do not give enough detail and they [authors of systematic reviews] are not able to decipher between male/female, sometimes even age”* (Interview #3: participant/ research collaborator).

Reporting research participant details using a social determinants of health framework was recommended as a helpful way to communicate differences in effect and facilitate understandings of how individual characteristics, such as gender, factor into differential effects of interventions. It is also important to understand how context (for instance, social, historical, political factors) might impact the effects of the intervention:

*“We often put…many of the characteristics that are in PROGRESS Plus [a social determinants of health framework] on our wish list to include them in our analyses but we’re so limited - in terms of barriers – limited by the data, there’s so much missing”* (Interview #8: researcher).

*“For example, with the elderly, I’ve read different definitions, Some will say over 55 or 65, 75 - so if they [researchers using reporting guidelines] could clarify the age because depending on the definition there will be other, more considerations as people age, of course there are just more co-morbidities and different issues to consider”* (Interview #3: participant/research collaborator).

#### “Implications of equity for sampling and analysis”

Participants indicated the need for the extension of the CONSORT Statement to accommodate statistics that communicate important information about health equity (i.e. they also emphasized the importance of reporting on subgroup analyses to assess health inqualities). Many participants indicated that it was important to encourage researchers to report on features of their studies that relate to health equity and to use statistical methods to assess diversity:

“*So sample size is obviously important if you want to know if equity is only coming into your study through subgroup analysis [then] it’s important that you indicate that you have enough sample – you know we often discuss sample size in terms of our overall study so we don’t really talk about how, how well powered the study is for subgroup analysis. I think sample size is somewhere it could be particularly important to discuss equity”* (Interview # 5: researcher, editor).

*“Because often you are looking for a more homogenous group [in non-health equity trials]. If you’re looking for a more diverse group then that might impact your sample size. You probably need to put some consideration into that when you report”* (Interview #6: researcher, ethicist)

A few participants indicated that there is a need to report on whether the subgroup analysis is pre-planned and to ensure that these subgroup data are analyzed appropriately:

*“I guess for statistical methods…one would certainly need to highlight what if any subgroup analysis will be done and to state it a priori. The other thing is maybe even a comment about missing [data] for the different groups. Sort of being transparent about it as an issue for, say, particular groups”* (Interview #8: researcher).

*“I think statistics are statistics and, you need to – you might be looking at more subgrouping there but it’s just - I don’t see any change [in the CONSORT Statement checklist]”* (Interview #13: policy/decision maker).

#### “Think beyond the immediate trial”

All participants discussed the importance of considering the potential impacts of randomized trials. Participants discussed the extension of the CONSORT Statement for equity as having impacts on the future conduct and use of research:

*“I think that the details are very, very important and I think that any rationale that a researcher has… for including or excluding [study details] really gives good contextual information to the results. Because then it gives a lot of information…people aren’t writing for today, they’re writing for 5 years from now”* (Interview # 2: researcher, participant/ research collaborator).

*“People might read that and think that applicability – is it applicable to my patient with stroke as opposed to, you know, applicable to all patients with stroke living in urban/rural, rich/poor, whatever the case might be”* (interview #11: researcher).

Some participants discussed the fact that greater reporting of detail relative to health equity through the CONSORT Statement would contribute to the synthesis of randomized trials and also build evidence for interventions with impacts on health equity:

*“An essential thing to report is how you have measured this social factor for which you are now reporting differential results. I mean – measures of socioeconomic status there are, of course, an almost infinite number of different ways which one could do that and if you’re trying to – in order to interpret – I would want to know in synthesis how that had been determined”* (Interview #7: researcher).

*“Everything, of course, should be explicit and as clear as possible and then identify what was not done…there are some wonderful studies, and then there’s sloppy studies. And, then when we get to the systematic review level we see [that] some of the carelessness has ramifications”* (Interview # 10: researcher).

As well, some participants expressed concern about potential intervention harms (including the exacerbation of inequity) and the importance of reporting this:

*“They might actually be perpetuating inequity that we see in clinical practice because we simply don’t have enough information on the effect of certain interventions across different groupings”* (Interview #2: researcher, participant/research collaborator).

*“I do trials with [devices] and it turns out that [these] interventions can only be effectively applied with people who already own [devices]. Because there is no way on a pragmatic level you will distribute [devices] to everybody. So by definition the intervention goes only for those who already own [devices] and if there’s a benefit to that intervention, as there often is, then you’re creating divide between those who own [devices] and those who cannot benefit from the interventions. So in some instances that kind of intervention can be considered as creating an inequity and it would be a relevant unintended effect to be reported”* (Interview #9: reasearcher).

In summary, the key informant interviews were used to elicit detailed perspectives from knowledge users on ways to improve the reporting of intervention effects related to health equity in randomized trials. The key informant interviews identified important concepts that are not yet well articulated in the literature and that were not generated in our other studies. The key informant interviews also allowed the expression of views and sharing of knowledge by those who have direct experience in trials as participants, to expand thinking about the implications of randomized trials in relation to health equity. The interview findings were used to identify real-life issues and concerns and to stimulate discussion about whether and how the CONSORT Statement might be extended for equity in feasible ways.

## Discussion

There were seven new themes identified that were not previously found during other research activities underway to develop CONSORT-Equity 2017: “Differentiate the type of trial”, “Prompts for health equity”, “Ethics matter”, “Describe unique research strategies”, “Clarity of reporting”, “Implications of equity for sampling and analysis”, and “Think beyond the immediate trial”. These themes and key informant data contributed to the broader CONSORT- Equity 2017 research agenda. The concepts identified in the key informant interviews were subsequently affirmed during the international online survey and in the face-to-face consensus meeting held to debate extension of the CONSORT Statement for equity. For example, the focus on ethics that was identified by all key informant interviews led to the addition of a new item on ethical concerns in CONSORT-Equity 2017. Furthermore, 16 items were identified for a health equity extension from key informant interviews (Table 4). The key informant interviews prompted the inclusion of these new concepts about health equity in relation to randomized trials, and the value-added by the key informant interviews to CONSORT-Equity 2017 affirmed the use of an integrated knowledge translation approach in guideline development.

The key informant interviews were conducted with those for whom CONSORT-Equity 2017 might be relevant and are an example of integrated knowledge translation (KT). The approach used in this qualitative study is congruent with and complementary to the broader integrated KT research approach used in the series of studies to develop CONSORT-Equity 2017 and in which knowledge users had been partners in the design, conduct, analysis, and reporting of the research studies. Integrated KT is an approach to research that involves engaging knowledge users (those who can act on research findings) in the research process – from defining the research question to the application of findings [14]. This approach to research is undertaken with the expectation that the outputs of the research will be relevant, useful, and useable; that is, more likely to be applied in practice and policy and, create greater capacity to use research by knowledge users [12, 21].

Participants who identified as participant/community collaborators provided new insights and clarity on what matters to patients and the public, in relation to health equity and randomized trials: concerns about the use of standard ethical procedures as a barrier to inclusive research conduct (see theme “Ethics matter”), how research methods could themselves be used to promote equity with new approaches to research (see theme “Describe unique research strategies”), a need to include and report clearly on populations that are not often included in randomized trials (see theme “ Clarity of reporting”), and to consider the longer-term impacts of the CONSORT Equity 2017 extension for future conduct and use of research (see theme “Think beyond the immediate trial”). These contributions introduced ideas and perspectives (forms of knowledge) that had not originally been anticipated in our study and that led to insights and discussion in subsequent stages of the larger research study and eventually changes in the reporting guideline (see Table 4). The use of semi-structured interviews provided the opportunity for knowledge user participants, including patients - a group not before included in reporting guideline development – to work with peers in research systems and to include their views. Our study is an example of a way that forms of knowledge from patient groups can be incorporated in such research, giving them the opportunity to engage in the development of data that incorporated their views directly into the work done to develop CONSORT-Equity 2017. The approach to engage knowledge users as partners throughout the process was a way to foster thinking and action for the inclusion of perspectives that included key informants in a previously established process of guideline development.

Our work promotes a structured and evaluative approach to the use of key informant interviews in guideline development. In a review of all other existing and proposed CONSORT Statement extensions we found that a similar approach was only used in one extension (CONSORT Statement extension for herbal interventions). In this extension, telephone interviews were conducted as part of a three-stage guideline development process, in which there was pre-meeting item generation and suggestions for revisions to the existing CONSORT Statement in preparation for the consensus meeting and then post-meeting feedback. However, the conduct of a qualitative study was not reported therein [22].

In our study, the key informant interviews facilitated collaborative engagement and involvement with knowledge users, and created opportunities for different knowledges to coexist within an established and academic approach to guideline development. Approaches to the engagement of participants/community collaborators in randomized trials and in particular the development of reporting guidelines are not yet well established, and this study can provide an example. It is important that a culture of inclusion is fostered in the reporting of research: we encourage those in research systems as well as knowledge users themselves to recognize the value and feasibility of knowledge user inclusion that includes patients.

Our process of guideline development used an integrated KT approach, affirms knowledge user engagement in the established guideline development process [22] and is expanded to include knowledge users who are also patient consumers (participants/research collaborators) of the evidence reported by CONSORT-Equity 2017. The key informant interviews were an integral feature of the guideline development process, and served to identify the real-life application of equity-related concepts generated within the context of randomized trials.

### Limitations and Strengths

One limitation of this study is that we engaged with a small and diverse group of key informants with 3 of the 13 identifying as participant/community collaborators (people who are patients and who are active in research), and the remainder consisting of a funder, ethicist, researchers, publisher/journal editors, and policy maker. As well, the sample of participants was biased in favour of researchers as users of the CONSORT Statement rather than community members or members who identified as part of a group that experiences disadvantage within health systems, a reflection of a broader research environment in which there is extremely limited reporting of patient engagement and involvement in randomized trials [23]. In addition, the study used qualitative methods that are not well established in guideline development (that is, the use of key informant interviews).

This study had several strengths. For example, the key informant study (from design through to the conduct and interpretation) involved an international and inter-disciplinary study executive and advisory board and so reflected a broad array of views that includes members who identify as part of a group that experiences disadvantage within health systems as well as members of other knowledge user groups (funders, editors, ethicists et cetera). While there was an in-depth focus on those who are known users of CONSORT Statement as a reporting guideline, we expanded the definition of knowledge user to include those who are also consumers (research participants who are patients, community members who choose to collaborate in research) of the evidence reported by CONSORT-Equity 2017. This meant that there was purposeful recruitment of research participants/community collaborators to ensure consideration and incorporation of their views. We were striving to introduce participation of patients in an area for which there is limited guidance and reported involvement and sought additional participant/community collaborators without success. However, the participant/community collaborators we did engage made substantial contributions to the study and demonstrated to all that participant/community collaborators can play a valued role, and in a way that is feasible to incorporate into established guideline development processes. As there is ‘road-testing’ of the CONSORT-Equity 2017 planned as part of the guideline dissemination [11] there are further opportunities for involvement and engagement that leads to feedback on CONSORT-Equity 2017 by knowledge users. Our use of key informant interviews was used to integrate real-world experience in health systems and contributes to the development of knowledge about a research guideline and demonstrates that there is an important role for knowledge users that include patients in the guideline development process.

Another strength is that the approach used in the key informant interviews accommodated the socio-cultural context of those participating in the interviews and maintained the fidelity of the CONSORT Statement. Two researchers were involved in the data coding and thematic development and there was member-checking of interviews for accuracy. The study executive members who were involved in the study had previous experience with the extension of other statements [24].

Finally, a consensus meeting attended by a broad range of potential knowledge users from high, middle and low income countries, including patients and methodologists [11, 25], affirmed the relevance of the key informant themes to the development of the CONSORT-Equity 2017 as a reporting guideline.

## Conclusion

The CONSORT-Equity 2017 statement promotes the reporting of factors that relate to unfair and avoidable differences between population groups. To help extend the CONSORT Statement for equity, we interviewed key informants who are potential CONSORT-Equity 2017 users to elicit knowledge user perspectives on the ways to improve reporting of intervention effects related to health equity in randomized trials. We used key informant interviews to obtain a detailed assessment of knowledge user perspectives and to identify ways to improve reporting of intervention effects related to health equity in randomized trials. We have described the findings from the key informant interviews on items for reporting to assess the effects of an intervention on health equity, and how the findings from key informant inteviews were used to develop a reporting guideline. We encourage the use of key informant interviews to engage and involve all knowledge users, including patients, to ensure the inclusion of real-life expertise in guideline development.

## Abbreviations

CONSORT: CONsolidated Standards Of Reporting Trials Statement
KT: Knowledge translation
